# Experiences of a Novel Structured Foot Examination Form for Patients With Diabetes From the Perspective of Health Care Professionals: Qualitative Study

**DOI:** 10.2196/45501

**Published:** 2023-07-18

**Authors:** Susanne Andersson, Isabella Scandurra, Ulrika Nyström, Marika Varemo, Ulla Hellstrand Tang

**Affiliations:** 1 Department of Health Sciences University West Trollhättan Sweden; 2 Centre of Empirical Research in Information Systems Örebro University Örebro Sweden; 3 Health Centre Dagson Uddevalla Primary Care Västra Götalandsregionen and Municipal Care Trollhättan Sweden; 4 Department of Medicine Northern Älvsborg County Hospital Trollhättan Sweden; 5 Department of Prosthetics and Orthotics Sahlgrenska University Hospital Gothenburg Sweden; 6 Department of Orthopedics Institute of Clinical Sciences, Sahlgrenska Academy University of Gothenburg Gothenburg Sweden

**Keywords:** diabetes, foot ulcer, prevention, primary health care, qualitative research, structured foot examination, validation, user experiences, participatory design

## Abstract

**Background:**

Diabetes is a growing threat to public health, and secondary diseases like foot complications are common. Foot ulcers affect the individual’s quality of life and are a great cost to society. Regular foot examinations prevent foot ulcers and are a recommended approach both in Sweden and worldwide. Despite existing guidelines, there are differences in the execution of the foot examination, which results in care inequality. A structured foot examination form based on current guidelines was developed in this study as the first step toward digitalized support in the daily routine, and was validated by diabetes health care professionals.

**Objective:**

The study aimed to validate a structured foot examination form by assessing health care professionals’ experiences of working with it “foot side” when examining patients with diabetes.

**Methods:**

Semistructured interviews were held in a focus group and individually with 8 informants from different diabetes professions, who were interviewed regarding their experiences of working with the form in clinical practice. The users’ data were analyzed inductively using qualitative content analysis. The study is part of a larger project entitled “Optimised care of persons with diabetes and foot complications,” with Västra Götaland Region as the responsible health care authority, where the results will be further developed.

**Results:**

Experiences of working with the form were that it simplified the foot examination by giving it an overview and a clear structure. Using the form made differences in work routines between individuals apparent. It was believed that implementing the form routinely would contribute to a more uniform execution. When patients had foot ulcers, the risk categories (established in guidelines) were perceived as contradictory. For example, there was uncertainty about the definition of chronic ulcers and callosities. The expectations were that the future digital format would simplify documentation and elucidate the foot examination, as well as contribute to the accessibility of updated and relevant data for all individuals concerned.

**Conclusions:**

The foot examination form works well as a support tool during preventive foot examination, creates a basis for decision-making, and could contribute to a uniform and safer foot examination with more care equality in agreement with current guidelines.

**Trial Registration:**

ClinicalTrials.gov NCT05692778; https://clinicaltrials.gov/ct2/show/NCT05692778

## Introduction

### General Background

Among persons living with diabetes, it is estimated that 5% have a foot ulcer [1-5]. This means that 23 million persons (5% of 537 million people with diabetes) at the global level [6] and 25,000 persons in Sweden have a diabetic foot ulcer (DFU) [7]. A large increase in people diagnosed with diabetes (increase to 783 million people) is expected by 2045 [6].

Groups with a low socioeconomic status have an increased risk of DFU, and men are more frequently affected [8-10]. A delay in detecting persons at risk of developing a DFU and a delay in access to treatment increase the risk of further complications, such as severe infections, leading to amputations [11].

The consequences of delayed treatment and a lack of foot screening are important for the individual and society. The quality of life of the individual is reduced in the presence of a DFU, and the costs are considerable [12-16]. The structure of health care differs between countries, as does the pathway toward prevention, and as a result, the treatment costs for DFUs vary among high-, middle-, and low-income countries [17]. Nevertheless, the impact on individuals in terms of their psychological well-being and their private economy is significant. The impact varies depending on the organization of health care in each country. In Sweden, health care is financed by taxes, and the 21 independent regions are responsible for delivering equal care of a high standard [18].

With effective prevention and structured processes among primary care, specialist care, and municipal care, DFUs can be successfully prevented [19-23]. Of the 500,000 persons with diabetes in Sweden, the majority are treated in primary care [7]. Promising examples show that, by using a structured standardized routine for foot assessment and risk stratification, greater quality of care is achieved [24,25]. In contrast, the lack of a structured foot assessment might lead to subjective risk stratification and inappropriate or nonactive consequences [26,27].

**Figure 1:**
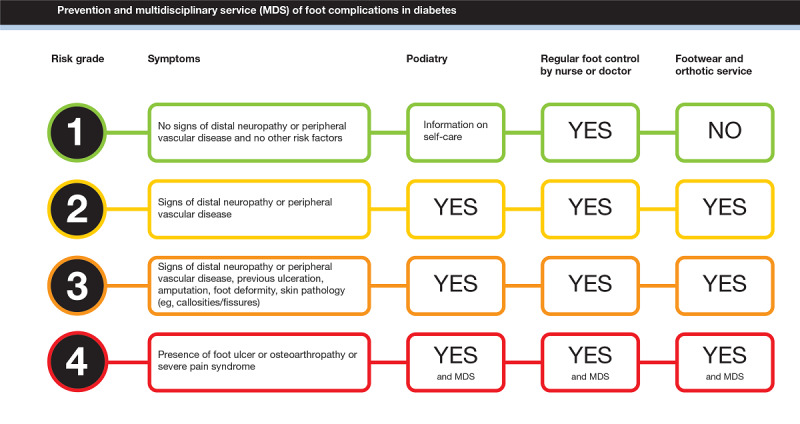
A scheme of the risk categories, symptoms, and recommended actions to prevent diabetic foot ulcers according to the national guidelines. MDS: multidisciplinary service.

National and international guidelines recommend that an annual foot examination should be offered to all patients with diabetes [28-30]. However, figures from the Swedish Diabetes Register show that the feet of 25% of people with diabetes were not examined in 2022 [31]. Traditionally, in Sweden, patients’ feet are examined at the same time as the annual diabetes examination in primary health care or at a clinic of medicine in specialist care. The examiner could be a physician, nurse, or podiatrist. After the examination is completed, the health care professional (HCP) should register in the Swedish Diabetes Register (1) that the person has undergone examination of the feet and (2) the risk grade of the person from 1 to 4 (1 [no risk of developing a DFU] to 4 [ongoing DFU]), with subsequent actions based on the risk category (Figure 1) [28]. The risk grade is based on findings of peripheral neuropathy, for example, identified with a monofilament test or the Ipswich Touch Test [32]; peripheral angiopathy; foot deformities; skin pathologies; foot ulcers; and previous foot ulcers/amputations [29].

### Study Background

Patient representatives and HCPs found it necessary to improve the routine of the annual foot examinations for persons living with diabetes in Västra Götaland Region (VGR), with the aim of enabling all patients with diabetes to have their feet examined in *a structured uniform manner*. As a result, a novel structured routine in paper format, a paper form, was developed and tested by persons with special competence in the area (Multimedia Appendix 1). This paper form was suggested as an initial tool to help HCPs perform annual foot examinations, as recommended in national guidelines [28]. Adhering to participatory design (PD) principles, the research team was aware that the focus of PD is not only the improvement of the information system but also the empowerment of workers, so they can co-determine the development of the information system and their workplace [33]. For this reason, before the form is designed for creating an existing or future clinical decision support system (CDSS), it needs to be validated by real users, that is, the HCPs performing the actual foot examinations in primary care.

In a previous project in the same region, VGR, a pilot test of a digital prototype was performed by a certified orthotist and prosthetists. It was shown that, by using a structured eHealth solution, the foot assessment and the subsequent automatically generated risk category produced a reliable uniform assessment, thereby facilitating documentation [26].

### Study Aim

**Figure 2:**
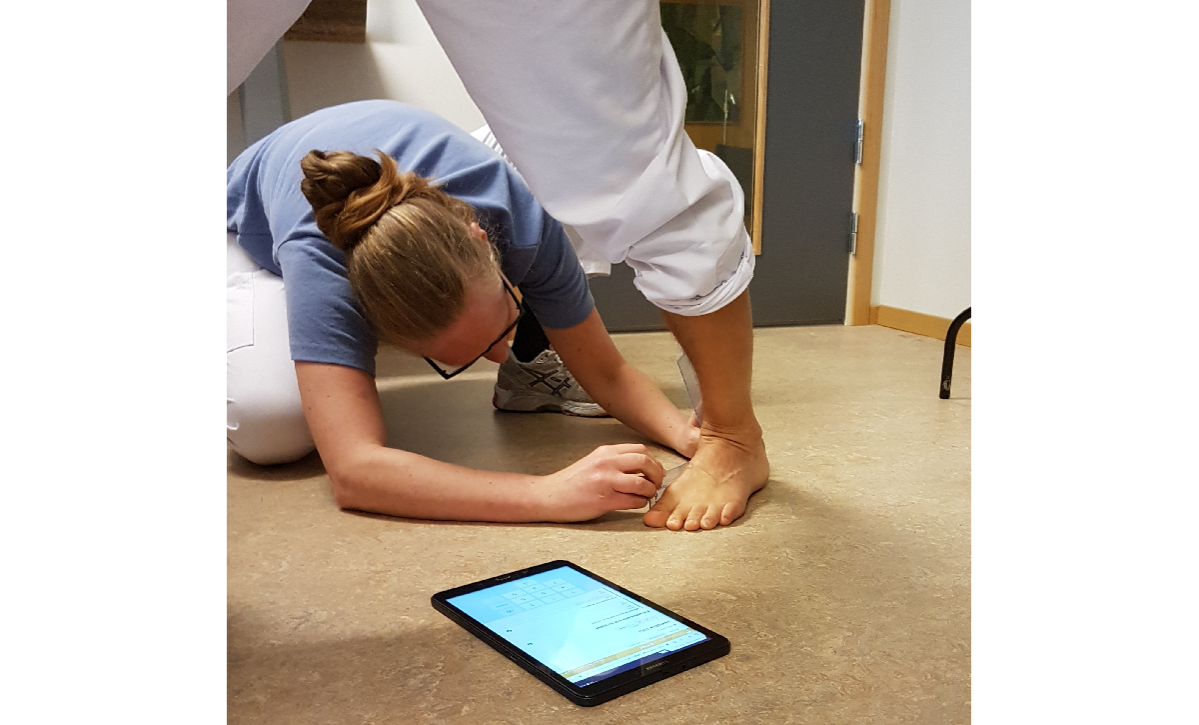
A health care professional measuring the passive range of motion at the ankle joint by using a goniometer close to the patient. The findings of the foot examination are registered on a tablet following a structured foot examination.

Eliciting future users’ real experiences is crucial in user-centered design (ISO 9241-210:2019) [34]. User experiences are differentiated from opinions, which could be held by designers, managers, or other secondary stakeholders of a system, as well as future users of that system. User experience is defined as “users’ perceptions and responses, including the users’ emotions, beliefs, preferences, perceptions, comfort, behaviors, and accomplishments, that occur before, during, and after use,” and it is related to “the context of use” [34]. Here, the context of use often means *on the floor*, close to a sitting patient, where the HCP examines the feet using various techniques and tools (Figure 2). It is therefore important to validate not only the information in the new tool but also the fact that novel tools can actually succeed in the clinical work situation of an HCP. To prepare for a digitally supported routine of a structured foot examination in an annual foot assessment, the structured routine was validated by real users, that is, HCPs in daily practice, performing daily work tasks on the floor. This study aimed to describe HCPs’ experiences of a novel structured foot examination form on paper (Multimedia Appendix 1) when performing annual foot assessments in patients with diabetes.

## Methods

### Design

The study is part of a larger regional project designed to optimize the care of persons with diabetes and foot complications. In this study, a qualitative descriptive approach was used to sum up the empirical data inductively in order to produce a common understanding that relates to general conditions within this context.

### Context

The study took place in 5 primary health care centers and 3 hospital care centers located in southwestern Sweden. To optimize a digital routine, the form in a paper routine (referred to as “form”) was used and tested by professional caregivers (Multimedia Appendix 1). The participants in the study were HCPs who usually examine the feet of individuals with diabetes, and they were strategically invited to test a form for annual foot examination. A total of 16 HCPs were asked to participate. Among these HCPs, 12 registered an interest in participating in the project and 8 were finally included. Three declined when asked to participate in the interview study and 1 person did not respond. Among the 8 participants, 3 were district nurses/specialist diabetes nurses, 3 were podiatrists, and 1 was a physician. The dropout resulted in a lack of nurses working in municipal care.

### Data Collection

Semistructured interviews were held in March 2021 with 2 focus groups and 2 individual interviews including 8 respondents from different professions within diabetes care (Table 1).

The respondents were interviewed about their experience of examining the feet of patients with diabetes according to the new form (Multimedia Appendix 1). The respondents had each used the form on at least five patient visits. The interviews took place digitally where the interviewers and respondents could see and hear each other during the interviews, apart from one individual interview where only sound was recorded. The interviews lasted between 30 and 90 minutes, and were recorded digitally, listened to several times, and transcribed verbatim by 2 authors (UN and MV). Open-ended questions regarding the experiences of working with the form in clinical practice were addressed and a semistructured interview guide was used (see examples in Multimedia Appendix 2). The opening question was “How comfortable do you feel about using the paper form in routine work?” Examples of other questions were “What were your expectations of the effectiveness of the form?” “What were your expectations of the degree of effort?” “How well does the form correspond to the expectations/help you to achieve the goal of the foot examination?” and “What is important to you?” (for further questions, see Multimedia Appendix 2). The interview guide ensured that a certain area of questioning was covered [35].

**Table 1:** Participants’ profession, context, and experience of diabetes care.

Participant	Profession	Context	Experience of diabetes care (years)
A	Nurse with an additional course in diabetes	Primary health care	1
B	District nurse/specialist diabetes nurse	Primary health care	1
C	District nurse/specialist diabetes nurse	Primary health care	7
D	District nurse/specialist diabetes nurse	Primary health care	17
E	Physician, internal medicine	Hospital care	15
F	Podiatrist	Primary health care	20
G	Podiatrist	Hospital care	20
H	Podiatrist	Hospital care	11

### Data Analysis

The qualitative research tradition is based on an effort to develop an understanding of the human lived experience. Qualitative content analysis, as described by Graneheim and Lundman [26], was considered appropriate in order inductively to focus on and describe experiences and variations on an individual level and to identify differences and similarities on a manifest and latent level. The analysis was conducted in several steps. First, all the data were read several times to ensure immersion in the data. All interview text was regarded as a unit of analysis. The text was then divided into “meaning units” that corresponded to the aim of the study. Each meaning unit was condensed and labeled with a code. Codes are described as concrete and as close to the text as possible [36]. During this process, the authors (SA, UN, and MV) continuously returned to the original text to ensure that the core meaning of the meaning units was maintained. Similar codes were later grouped into subcategories, and sorted and abstracted into higher categories (Table 2).

Finally, the latent interpretative analysis was described as the main theme with a higher level of interpretation and abstraction.

A continuous verification of the interpretation was an ongoing process during the search for coherence among the different parts of the analysis. To ensure trustworthiness, there was an awareness of and an openness toward misunderstandings resulting from the interviewer’s own preunderstanding. To avoid misunderstandings, the data were first analyzed individually and later compared by 3 researchers.

**Table 2:** Analysis process example.

Meaning unit	Condensed meaning unit	Code	Subtheme	Theme
The expectations are...it should be a form that covers the area of feet and diabetes...can be read regardless of who has filled it in so you get an overview.	Easy form that completely covers the area relating to the feet, regardless of who fills in the form.	Easy form, covering the area relating to the feet in full.	The structure is clear and comprehensive.	Structured support
When I received the form, there was a lot of text to take in/…then I realized that we do these surveys, or have always done them during an annual check-up.	Lots of text to absorb, then I realized that we have always done it.	We have always done it.	This is what we are already doing, with some new elements.	Structured support
That we all do the same thing is very good both for us, if we take over other patients, or when the doctor conducts the annual check-up, so that they see exactly what it is we have assessed and looked at.	That we all do the same thing is good when we take over each other’s patients so they see what it is we have assessed.	Everyone makes the same assessment.	Support for a standardized survey.	Structured support

### Ethical Considerations

The study was approved by the Swedish Ethical Review Authority (diary number: 2020-02715 and 2020-05131) and was conducted according to the ethical principles described in the Helsinki Declaration [37]. All participants were informed, both verbally and in writing, about the study before obtaining their written informed consent. The participants were ensured confidentiality and were free to withdraw at any time. The transcripts were anonymized and given a letter (A-H), which was subsequently used in the analysis. The possible harm caused by the study was evaluated against the benefits. The participants were informed that any concerns could be clarified by contacting the authors. No such concerns were raised.

## Results

### Support of Professional Judgement

The results at the highest level showed that the participants felt that the theoretical support provided by the form was needed (Multimedia Appendix 1), but it also had to be verified by the HCP’s judgement, experience, and knowledge, which was reflected in the practical implementation. The main theme was “structure and documentation as a support for professional judgement” (Table 3). Three themes were identified: (1) structured support, (2) professional evaluation is needed, and (3) documentation that simplifies and makes visible, referring to both patients and their feet. Multimedia Appendix 3 presents some examples of expressions that are cited in this study in Swedish and English.

**Table 3:** The subthemes, theme, and main theme emerging from the analysis.

Subthemes	Theme	Main theme
This is what we are already doing, with some new elements The structure is clear and comprehensive Support for a standardized survey	Structured support	Structure and documentation as a support for professional judgement
Risk rating simple – contradictory Some steps may be evaluated	Professional evaluation is needed	Structure and documentation as a support for professional judgement
The form makes the feet visible Facilitating documentation provides time for the patient meeting Easier with a digital format	Documentation that simplifies and makes visible	Structure and documentation as a support for professional judgement

### Structured Support

The theme of structured support included the respondents’ experience of the structured foot examination protocol and that it offered structure, clarity, and support in performing foot examinations.

#### This is What We are Already Doing, With Some New Elements

Initially, the form was perceived as rich in text, which required limited reading before the HCPs felt familiar with the structure. After practicing it in several examinations, they said that they learned the structure. New routines always require a period of practice, as it is a new way of doing things. One respondent commented:

The very first time, I was like this, wow! What shall I do? There is so much text before you get into what it really said.

Respondent D

The informative text, which was first perceived as compact, became helpful and supportive. The respondents said that the form was effective and useful as a basis for the annual foot examination after a running-in period.

The results showed that the steps and parts included in the form were largely familiar, were well known, and had already been performed every year in previous foot examinations. The form covered what was perceived as important, the very essence of the foot examination, according to existing guidelines. Respondent D commented, “This is definitely back to basics.” Although the form contained much that was familiar, the examination with the Ipswich Touch Test was new [32]. However, as the test was used with the structured examination, it was perceived as a simple useful test.

#### The Structure is Clear and Comprehensive

The form enabled an overview of the feet and was experienced as a supportive and comprehensive guide for the foot examination. The content was divided in a clear distinct way and could be read by everyone, which increased the common understanding between the different caregivers. They said that the form contributed to an evidence-based foot examination*.* Respondent A commented, “So I think it’s very clear and very good, because it’s safe.” The 3 areas of the structured foot protocol were described as being composed in a structured and appropriate manner.

It is structured in such a way that there are three parts, inspect and examine, palpate and examine and symptoms and previous ulcers. It is a very good and very logical division based on the different parts.

Respondent F

The experience of the form was that it was easy to use and follow, and it was a well-functioning basis for the preventive foot examination and an aid for those who worked in primary care and in specialist care. It was also described as something that could easily be used in daily patient work. The structured form was clearly designed and described as user friendly. The proposals for action on the back of the form were described as clear and good (Multimedia Appendix 1).

#### Support for a Standardized Survey

The form could be a support for examining feet for both beginners and those who are more experienced. It served as a support and provided new knowledge about the foot examination and subsequent measures for those who used the data. Even for experienced professionals, it could be useful as a basis for decision-making and documentation.

Sometimes I think things have been a bit fluid at the health center, that they have sensory impairments, but they do, so it was not clear what I would do when I found this. So I thought it would be nice to get a little more knowledge about foot examinations.

Respondent A

Although the form provided support for the implementation of the foot examination in various ways, there was also experience that the form was not needed as support. This was related to the fact that the participants already had a good structure to follow and had extensive experience in the field.

You check it anyway, so, for my part, I don’t think I need to have something like this to do the job I do now.

Respondent H

The use of the form highlighted the fact that foot examinations were performed in different ways by different HCPs at the same unit. Having a form to follow could contribute to the standardization of the foot examination. One respondent commented:

We realized quite quickly that everyone does things very differently and everyone has learned differently, too, so that you then have some form of action, that everyone does the same thing, which can be good.

Respondent A

After the introduction of the form, collaboration between colleagues was facilitated. The form was stated to structure and align the foot examination, which was thought to be able to lead to more equal care regarding the prevention of foot complications. This meant that the form was seen as useful and adequate in the work on foot examinations in patients with diabetes.

### Professional Evaluation is Needed

The theme includes the need for professional judgement to evaluate certain aspects of the foot examination. Judgement was needed whenever the form was used, from the interpretation of the risk categories and different concepts to the actual conduct of the examination.

#### Risk Rating Simple – Contradictory

The experience of grading in the form was that it was confusing at first, but it became easier after conducting some surveys. There was some difficulty owing to contradictory risk categories, as foot ulcers could be classified as risk category 4, and subsequently, the patient might have risk category 1, which indicates a healthy foot.

It’s as though you are talking against yourself when you put a four, because it is such a high degree of risk and then you have to put a risk category, 1-4 to the right there for the others and we didn’t think that was easiest approach. Then, all of a sudden, you write that it is a healthy foot.

Respondent G

Although it was always the highest figure that gave the final risk category, the grading involving assessment points was illogical and contradictory, which made the grading itself difficult to understand, but it was otherwise easy to follow the form.

That was what we brought up, it was the grading that was a bit strange, so that’s it. Otherwise it’s not that strange, it’s just reading.

Respondent H

It was said that a healthy foot does not involve any risk and thus should be classified as risk category 0 and not risk category 1, but this was not perceived as an obstacle to continued use, because the participants quickly changed their minds and accepted risk category 1 as the lowest risk. After all, the HCP was the one who filled in the highest risk category in the summary box, which was identified during the examination.

The form simplified the documentation because the user could just tick and get a number for the risk category, instead of writing everything from the foot examination in free text as before. The number system for risk category (Figure 1) was said to be simple and good, with figures that corresponded to the various risks in a fair way. A number system, defining the risk categories, could also make the assessment clearer and could be reproduced in a simple way from one examination to another.

Some aspects were clinically difficult to assess, such as what was considered hardening skin (calluses), an ulcer that was difficult to heal, or reduced hair growth. The definitions for hardening skin and healing ulcers, and how medical records and patient narratives could be used to assess previous ulcers and reduced hair growth were discussed. The idea was raised that it might be valuable to line up ulcers of different types in categories. Ulcers were identified as serious, and it was therefore good that this was first on the form.

#### Some Steps May Be Evaluated

Professional judgement is used in different ways and in different parts. There was difficulty conducting the examination in some patients with dementia, for example, and uncertainty about whether the patients understood the given information and provided correct information regarding symptoms, especially emotional disorders. At the same time, the experience was that these difficulties have always existed and that you then had to do your best and make a professional assessment or estimate, but that the form still worked well in most cases.

No, I think that the form feels, as I see it anyway, completely correct, because, no matter how I say this, I have worked with … all the time and these difficulties with precisely these patient categories have existed all the time, so it’s nothing new, but you have to find a way to move forwards in some way. I’m just mentioning it as an observation.

Respondent B

The respondents’ experiences were that it could be difficult for patients to distinguish between the middle toes on the Ipswich Touch Test and that the test should not be performed too quickly, because the patient then would not have time to understand which toe was affected. During the study, some respondents made comparisons of different instruments to assess neuropathy to see if there was any difference in the assessment, for example, monofilament and the Ipswich Touch Test, but the conclusion was that the result was usually the same. A biothesiometer, an old instrument for measuring sensation, was used by some professionals before the Ipswich Touch Test. After a comparison, the respondents said that there was a difference in assessment between the Ipswich Touch Test, a tuning fork, and the biothesiometer, as they showed different results regarding the sensation in the foot.

### Documentation That Simplifies and Makes Visible

The theme includes the structured form being perceived as making visible and clarifying the importance of foot examinations for both the patient and HCP, and the documentation needing to be facilitated and made visible, with the hope of simplification in the future digital format.

#### The Form Makes the Feet Visible

During the foot examination, the respondents talked about the feet, and explained what they did, why self-care was important, and what patients could do themselves. The structure of the form helped to focus on the feet, highlighting existing guidelines. In this way, it served as an educational tool during the foot examination.

… these guidelines have been around all the time I have been working, I can say, and the latest, they are here and they say exactly the same thing, being able clearly to show the patient what the criteria were for medical foot care was described as important, so that it would not be interpreted as meaning that the assessment was arbitrary, as many patients wanted to receive foot care even though they did not meet the criteria, as it says on this form that we have here now, so I think by far the best thing is that it is noticed. That’s what happens, it is, made visible.

Respondent B

Self-care was described as being of great importance and an obvious part of the conversations with the patients, and the form could be a way of making visible the importance of self-care to prevent foot complications. Brochures or written advice on self-care were often distributed before, and written information in the form of a self-care brochure was seen as a good tool, provided that the patient had an interest.

What the guidelines were for foot examinations and what examinations were made during the visit were also made visible to other care providers and to the patient. The criteria for medical foot care, for example, were also made visible, and the form could be shown to justify why a referral was or was not written.

Being able to clearly show the patient what the criteria were for medical foot care was described as important, so that the assessment would not be interpreted as arbitrary, as many patients wanted to receive foot care, even though they did not meet the criteria.

If you have good sensation, then you can say it, but it’s great that you still have good sensation so that you don’t need medical foot care, you can show it and vice versa, if they really have reduced sensation and therefore need a referral to foot care, so that’s good.

Respondent E

From a resource point of view, it was said to be impossible for everyone who wanted medical foot care to receive it. It was therefore important that the referrals were demand driven and based on clear criteria.

#### Facilitating Documentation Provides Time for the Patient Meeting

The form was filled in during the visit or close to it. The experience was that the documentation was extensive, but adjustments were made so as not to disrupt the meeting and contact with the patient. One example was to write words of support on the form during the visit or after the examination, so the HCP could dedicate time to the patient, be present, and have eye contact. Documentation in the medical record was sometimes started during the visit due to lack of time, but this usually happened after the visit.

I document when I have the patient with me. I also have a piece of paper next to it where I also write some notes, because you want to have eye contact with the patient so that you have a dialogue. I do that…

Respondent C

Often, some own words were needed in addition to the form in order to remember and compare the foot status from time to time. This facilitated the memory image of the patient. It was very important for the form to facilitate documentation and not the other way around. The documentation must be effective and be made quickly.

So I feel that it is very important that the form is as easy to fill in as possible and that it is still as comprehensive regarding the foot, but it can be filled in quickly because you spend a lot of time on documentation.

respondent E

More time with the patient was important for a good meeting. A good form was therefore considered to be one that is comprehensive but at the same time flexible and easy to use, where the HCP does not have to write a lot.

Many patients are actually looking forward to this meeting and it’s important that it’s a good meeting and that the documentation can perhaps be straightforward.

Respondent E

#### Easier With a Digital Format

The results of this study include the evaluation of a form that can be implemented in a digital format. A request was made for the risk degree, risk category, and measures to automatically emerge when the assessment is made. It was also suggested that the mouse pointer could be placed at a certain point where it is possible to obtain more informative text, if necessary, to facilitate the overview, so that there would not be too much text in the form itself.

I have thought about it a bit and I wonder whether there shouldn’t be some kind of combi box, i.e. that you are able to choose alternatives that then fall into risk category one, two, three and four and if there are then measures that pop up automatically.

Respondent C

There were also suggestions that it would be easier with a digital format because the respondents wanted to avoid the double documentation that is thought to take place at present.

As it is now, we have paper when we do it and then we put it in the medical record. We do things twice now and, if it is possible to deal with everything once, it would be easier.

Respondent G

The form was perceived as a uniform assessment with everyone working in the same way. In the future digital format, expectations were expressed that increased collaboration would be possible between different care units, regardless of care organization; between colleagues; and between different staff categories in order to achieve a higher quality of care.

## Discussion

### Principal Findings

According to the respondents, there were expected results, as well as some unexpected findings. First, the most important finding was that adherence to clinical guidelines was facilitated using the form. Similar experiences were expressed by both experienced professionals and those with less experience. Second, when evaluating the risk of developing DFUs, professional judgement was emphasized by the respondents. Just filling in a paper form does not improve the quality of care, as stated by the respondents. Judgement was needed throughout the use of the form, from the interpretation of the risk categories and the different contexts to the actual conduct of the examination, as well as when dealing with follow-up actions. However, the form provided the necessary structure and support in the decision-making process.

By using the form, the respondents also found that they had a new educational tool in their hands. The form could be used as an incentive, for example, to clarify the different parts of the foot examination for the patient and why these parts were checked, and as a result, it became an educational tool. One unexpected finding was that the respondents stated that it felt natural to use the form to initiate conversations about self-care, medical foot care, any need for insoles, and advice on choosing shoes. By creating processes that lead to the patient becoming involved, the potential for autonomy and self-determination also increases for the patient [38]. The form thus strengthens the patient’s participation and partnership, in line with person-centered care [39] and the theories of shared decision-making [40] and self-determination [41].

As part of the completed foot examination, the respondents also found that time to form a professional judgement was required to evaluate the results based on each person’s needs and to connect the results to the next activity in the care plan.

### Evaluation of Outcomes

After using and validating the form according to current work practices, some expected results were obtained, for example, the structure helped to perform the examination and the form also required professional judgement to be taken into account when formulating the individual results and needs into recommendations and further steps in the care plan. The respondents were surprised that the form was also suitable as an educational support tool when discussing self-care with the patient. This is often the case, and positive side effects like this emerge when users are allowed to handle novel tools or prototypes in their real work setting or in a user situation [42]. It is crucial for users to be involved at an early stage in the design process [34]. From the point of view of the PD method used, we could also see that these unexpected findings could be the subject of future studies and included in the design of the next stage of digital CDSS development.

Involving a user-centered design process, this study followed the basic steps where an early design should be tested in a realistic setting, even before software is developed. Previous research on the process of user needs and context requirement gathering reminds us that “actual requirements pre-exist our effort to capture them” [43]. This means that, as developers, designers, researchers, and other work process experts, we must be careful not to create requirements that fulfill only our own technical or organizational desires. This concept is particularly important in domains where users are less familiar with digital tools [44]. To resolve this issue, the validation process used in this study aimed to emphasize real needs, as expressed by real users. An understanding close to “best practice” will enable us to design and develop a digital tool and simultaneously assist in structuring and enhancing the work process of HCPs managing patients with diabetes. Moreover, the results here will generate a detailed context of use analysis, based on the experiences of real future users, which in turn will result in a shorter and less expensive development phase. For future iterations, the respondents who were active in this study have already volunteered for further work, knowing that their points of view will be included. Another strength here is that the validation was carried out by HCPs with both long-term and short-term experiences of foot examinations, who had different professions and worked at different care units (both primary care and regional care).

### Limitations

A qualitative method always involves subjectivity, as the researcher is part of the research. By being aware of one’s own preunderstanding, this issue can be managed throughout the process. During the analysis, a continuous dialog took place and a broader understanding was created to achieve a reliable result [45].

The dropout of 4 participants meant that no individual from municipal care participated. Thus, the transferability of the study results might not apply to all forms of care. One weakness of the study was that the use of the form was not very high, but the data that emerged during the interviews were perceived to be rich and to have a high degree of validity.

### Comparison With Prior Work

In line with previous studies, this study provided promising examples that the use of structured standardized routines for foot assessment and risk stratification can lead to higher quality of care [24,25]. The informants in this study stated that a structured routine would enhance care. Higher care quality for patients with diabetes and reduced health care costs are expected [13,46] after the early detection of risk factors, followed by rapid prevention, including a dialog on self-care, a referral to podiatry, and a referral for therapeutic footwear. Overall, the prevention of DFUs will preserve the quality of life of patients [24,25].

### Conclusions

The foot examination form works well as a support tool during a preventive foot examination. It offers a basis for decision-making and could contribute to a uniform safer foot examination with more care equality, which is consistent with current national guidelines. The form may need to be supplemented with functionality to increase usability in connection with foot examinations, that is, printing and handing over personally adapted self-care advice based on the results of the foot examination. All the respondents appreciated participation in the validation of a digital tool for enhancing their daily routine.

## Appendix

Multimedia Appendix 1 The structured foot examination in paper format.

Multimedia Appendix 2 Interview guide.

Multimedia Appendix 3 Examples of expressions that are cited in the current manuscript in Swedish and English.

## Abbreviations

CDSS: clinical decision support system
DFU: diabetic foot ulcer
HCP: health care professional
PD: participatory design
VGR: Västra Götaland Region
