# Supplementary material for: Experiences of a Novel Structured Foot Examination Form for Patients With Diabetes From the Perspective of Health Care Professionals: Qualitative Study
Source: JMIR Nurs. 2023 Jul 18;6:e45501. doi: 10.2196/45501 (PMC10488031; doi:10.2196/45501)
Supplement: Multimedia Appendix 1 [file nursing_v6i1e45501_app1.pdf]

Appendix 1. The structured foot examination in paper format

In Swedish:

Fotundersökning vid diabetes

**Riskkategori**	**Instruktioner**	**Riskgrad** om ja på frågorna	**Riskkategori 1-4** 1=frisk fot	
**Inspektera och undersök**			Hö	VÄ
Hudpatologier	Finns sår?	4		
	Tryckskada, sprickor, förhårdnader, liktornar, nagelproblem som kan medföra risk för sårbildning?	2		
Nedsatt blodcirkulation/ nerv-funktion	Finns torr hud, blek hud, hudtemperatur, svullnader, eller minskad hårväxt?	2		
Fotdeformitet	Finns amputation på tå-fot-ben eller hallux valgus, hammartår?	3		
**Palpera och undersök**				
Nedsatt blodcirkulation pulsar	Kan a. dorsalis pedis och/eller a. tibialis posterior **inte** palperas? (gå vidare med dopplerundersökning)	2		
Nedsatt nervfunktion ytlig	Har patienten nedsatt nervfunktion enligt Ipswich Touch Test/ monofilament?	2		
Nedsatt nervfunktion djup	Har patienten nedsatt nervfunktion testat med stämgaffel C128 Hz ?	2		
**Patientens symtom och tidigare sår**				
Nedsatt nervfunktion	Känner patienten pirrning eller domning/kuddkänsla i fötterna eller har en förändrad känsla i fötterna nu jämfört med tidigare?	2		
Tidigare sår	Har patienten tidigare haft ett svårläkt fotsår?	3		

Utifrån ovanstående undersökning och anamnes kategoriseras risken och definieras enligt nedan.

Högsta riskgraden skrivs in rutan ☐ Ange riskgrad (1-4)
Högsta riskgraden överförs med automatik till NDR

Har du haft samtal om egenvårdsråd, muntligt och skriftligt? ☐ Ja
Åtgärd dokumenteras i journalen

Åtgärder

Riskkategori 1 - Låg risk

Frisk fot – diabetes utan komplikationer:

1. Hälso- och sjukvården ska göra årlig bedömning av fotstatus utifrån fotundersökning och rapportera till NDR
2.Egenvårdsråd ges till patient. Informera patienten att ta kontakt med sjukvården om det sker en förändring på fötterna.
3. Skriftlig information om egenvård, till exempel Diabetesförbundets broschyr ”Fina fötter”, https://www.diabetes.se/contentassets/388af80eee2a462497ceae4fbe204342/fina-fotter---forebyggande-fotvard.pdf [
4. Egenvårdsråd samt självfinansierad fotvård och skor.

Riskkategori 2 Medelhög risk
Neuropati och/eller angiopati:

1. Hälso- och sjukvården ska göra årlig bedömning av fotstatus utifrån fotundersökning och rapportera till NDR
2. Egenvårdsråd ges till patient. Information om att ta kontakt med sjukvården om det sker en förändring på fötterna
3. Medicinsk fotterapeut bör kopplas in för förebyggande fotsjukvård
4. Ortopedteknisk kompetens bör kopplas in för att skydda fötterna från tryckskador.

Riskkategori 3 - Hög risk
Tidigare diabetessår, fotdeformiteter, förhårdnader, amputation:

1. Hälso- och sjukvården ska göra minst en årlig bedömning av fotstatus och utifrån fotundersökning med årlig rapport till NDR
2. Egenvårdsråd ges till patient. Information om att ta kontakt med sjukvården om det sker en förändring på fötterna
3. Fortsatt uppföljning efter läkt sår eller amputation hos patientansvarig läkare
4. Fortsatt uppföljning hos medicinsk fotterapeut
5. Ortopedteknisk kompetens bör kopplas in för att skydda fötterna från tryckskador.

Riskkategori 4 - Mycket hög risk
Pågående allvarlig fotsjukdom som sår, kritisk ischemi, infektion, Charcotfot:

1. Hälso- och sjukvården ska göra minst en årlig bedömning av fotstatus och utifrån fotundersökning med årlig rapport till NDR
2. Egenvårdsråd ges till patient. Information om att ta kontakt med sjukvården om det sker en förändring på fötterna
3. Uppföljning av fotsjukdom enligt landsting/regioners lokala rutiner
4. Fortsatt uppföljning hos medicinsk fotterapeut
5. Ortopedteknisk kompetens bör kopplas in för att skydda fötterna från tryckskador.

Om pågående allvarlig fotsjukdom upptäcks inom vården och omsorg, och tecken till läkning inte skett inom tio dagar – kontakta fotmottagning/multidisciplinärt fotvårdsteam för diskussion om fortsatt handläggning.

Appendix 1. The structured foot examination in paper format
In English:

Foot examination in diabetes

**Risk category**	**Instructions**	**Risk level if "Yes" to questions**	**Risk category 1-4** 1=healthy foot	
**Inspect and examine**			R	L
Skin pathology	Are there any ulcers?	4		
	Pressure sores, cracks, callosities, corns, nail problems that could result in a risk of ulcer formation?	2		
Reduced blood circulation/nerve function	Is there any dry skin, pale skin, skin temperature, swelling or reduced hair growth?	2		
Foot deformity	Is there any amputation on the toe-foot-bone or hallux valgus, hammer toes?	3		
**Palpate and examine**				
Reduced blood circulation pulses	Is it **not** possible to palpate a. dorsalis pedis and/or a. tibialis posterior (continue with a doppler exam)	2		
Reduced nerve function superficial	Does the patient have reduced nerve function according to the Ipswich Touch Test/monofilament?	2		
Reduced nerve function deep	Does the patient have reduced nerve function tested with a tuning fork C128 Hz ?	2		
**Patient's symptoms and previous ulcers**				
Reduced nerve function	Does the patient experience tingling or numbness/walking on pillows in their feet or a change of sensation in their feet compared with before?	2		
Previous ulcers	Has the patient previously had a foot ulcer that had difficulty healing?	3		

From the above examination and patient history, categorise the risk and define it as follows.

Put the highest risk level in the box ☐ Give the risk level (1-4)
The highest risk level is automatically transferred to the NDR

Have you spoken to the patient with advice on self-care, both verbally and in writing?

☐ Yes

Document the action in the medical record
