# Supplementary material for: Experiences of a Novel Structured Foot Examination Form for Patients With Diabetes From the Perspective of Health Care Professionals: Qualitative Study
Source: JMIR Nurs. 2023 Jul 18;6:e45501. doi: 10.2196/45501 (PMC10488031; doi:10.2196/45501)
Supplement: Multimedia Appendix 2 [file nursing_v6i1e45501_app2.pdf]

Appendix 2. Interview guide
The questions are presented in Swedish followed by the English translation

Hur **användarvänligt** upplever du att formuläret är/inte är? Vad i användningen är det lätta/svårare/omöjliga? **Tydligt eller ej?**

Did you experienced that the form was userfriendly/ not userfriendly? When using the form, what was easy/difficult/impossible. Was the form clear or not?

Var och i vilka miljöer används formuläret? Hur gjorde du? (fyllde i Foot Side eller på kontoret…?) Hur gjorde du när du dokumenterade?

Where were you when you used the form? In what context were you? How did you do? (filling in the form foot side or at the office…? How did you do when you made the documentation?

Vad har du för tankar om formuläret i pappersformat respektive **digitalt format**? Möjligheter/hinder? Idéer om hur överföring från pappersformat till digitalt skulle kunna ske? Vad har du för tankar kring eventuellt **elektroniskt verktyg** Foot Side? Vilka ska kunna nå informationen? **Återkoppling till patient?**

What are you thoughts regarding the form in paper form and in a digital form? Possibilities/hindrance? Ideas of how to transform the paper form to digital form? What are your thought regarding to use a digital eHealth tool Foot Side? What persons do you think should be able to see the information? Feed back to patient?

Kan formuläret fungera som **beslutsstöd?** Kan formuläret stödja patientens egenvård?

Could the form be seen as a **decision support system**? Can the form support the patients self-care?

Formulärets utformning? **Fattas något? Är något överflödigt?**

The design of the form? Is anything lacking? Can something be removed?

Hur ser du på ett **införande** av formuläret i journalsystemet? -Vad krävs? Vad skulle du önska? Har du de redskap som behövs för att utföra undersökningen enligt formuläret?

How do you think upon an **implementation** of the form in the electronical medical record system? – What is required? What do you wish? Do you have the tools that are needed as to perform the examinations according to the form?

När kan du tänka dig att delta i en motsvarande insats, fast med ett digitalt instrument?

When is it possible for you to participate in a similar test, testing a digital tool?

Är det något du vill ta upp som ännu inte framkommit?

Do you want to add thought that yet not have been addressed?

Vi vill tacka för ditt/ert medverkande!

We would like to thank you for your contribution.

Även "Utvecklingsteamet" hälsar och tackar!

Also ”the Development team” is greeting and says “Thanks”!

Om ni önskar kan ni få ta del av resultatet av studien kan ni kontakta XXX.

Om you wish to take part of the results of the study you can contact XXX
