# Supplementary material for: Experiences of a Novel Structured Foot Examination Form for Patients With Diabetes From the Perspective of Health Care Professionals: Qualitative Study
Source: JMIR Nurs. 2023 Jul 18;6:e45501. doi: 10.2196/45501 (PMC10488031; doi:10.2196/45501)
Supplement: Multimedia Appendix 3 [file nursing_v6i1e45501_app3.pdf]

Appendix 3. Examples of expressions that are cited in the current manuscript, in Swedish and in English

In Swedish	***Translated to English***
...allra första gången, så var jag så här whoo! Vad ska jag göra, vad mycket text det är liksom, innan man satte sig in i vad det egentligen stod.	*The very first time, I was like this, wow! What shall I do? There is so much text before you get into what it really said*
…Det här motsvarar definitivt back to basic.	*This is definitely back to basics*
Så det tycker jag är väldigt ja, tydligt och väldigt bra, för det är säkert.	*So I think it's very clear and very good, because it's safe*
…det är strukturerat på ett sådant sätt att om man tittar på det liksom, det är tre delar, Inspektera och undersök, Palpera och undersök och Symtom och tidigare sår. Det är en väldigt bra och väldigt logisk uppdelning av det utifrån de olika delarna.	*It is structured in such a way that there are three parts, inspect and examine, palpate and examine and symptoms and previous ulcers. It is a very good and very logical division based on the different parts*
Det har ibland tycker jag varit lite flytande på vårdcentralen, att men dom har känselnedsättningar men det har man, alltså, det var inte någon tydlighet vad jag skulle göra när jag hittade det här, så. Så jag tyckte att det skulle vara skönt, att få lite mer kött på benen inför fotundersökningar.	*Sometimes I think things have been a bit fluid at the health centre, that they have sensory impairments, but they do, so it was not clear what I would do when I found this. So I thought it would be nice to get a little more knowledge about foot examinations*
Det kollar man ändå så för min del tänker jag inte att jag behöver ha ett sånt för att utföra det jobbet jag gör nu	*You check it any way, so, for my part, I don't think I need to have something like this to do the job I do now*
vi insåg ganska snabbt att alla gör väldigt olika, och alla har lärt sig olika också, så att då har man ju någon form av, att alla gör likadant, vilket kan vara bra.	*We realised quite quickly that everyone does things very differently and everyone has learned differently, too, so that you then have some form of action, that everyone does the same thing, which can be good*
...det känns lite som att man talar emot sig själv när man sätter en fyra för det är ju en så hög riskgrad och sen ska man ju sätta riskkategori då, 1–4 till höger där på dom andra och det har vi väl vi tyckt inte har varit det lättaste. Så helt plötsligt skriver man att det är en frisk fot.	*It's as though you are talking against yourself when you put a four, because it is such a high degree of risk and then you have to put a risk category, 1-4 to the right there for the others and we didn't think that was easiest approach. Then, all of a sudden, you write that it is a healthy foot*
...det var ju det som vi tog upp det var ju graderingen som var lite konstig, så det är ju det, annars är den ju inte konstig, det är ju bara att läsa innantill.	*That was what we brought up, it was the grading that was a bit strange, so that's it. Otherwise it's not that strange, it's just reading*
…nämen jag tänker att formuläret alltså känns som jag ser det i alla fall helt rätt för att oavsett	*No, I think that the form feels, as I see it anyway, completely correct,*

så som jag säger det här har jag jobbat med hela tiden och dom här svårigheterna med just dom här kategorierna av patienter har ju funnits hela tiden så det är ju inget nytt så, utan man får hitta ett sätt att komma vidare i det på nåt sätt. Jag bara nämner det som ett observandum…	*because, no matter how I say this, I have worked with … all the time and these difficulties with precisely these patient categories have existed all the time, so it's nothing new, but you have to find a way to move forwards in some way. I'm just mentioning it as an observation*
…de här riktlinjerna har ju funnits hela tiden som jag har jobbat, kan jag säga och de senaste de ser ju ut, det är ju dom här och det står ju precis samma som det står på det här underlaget som vi har här nu, så jag tycker det absolut bästa det är att det blir lyft nu. Det blir liksom så, synliggjort.	*these guidelines have been around all the time I have been working, I can say, and the latest, they are here and they say exactly the same* thing, *being able clearly to show the patient what the criteria were for medical foot care was described as important, so that it would not be interpreted as meaning that the assessment was arbitrary, as many patients wanted to receive foot care even though they did not meet the criteria, as it says on this form that we have here now, so I think by far the best thing is that it is noticed. That's what happens, it is, made visible*
…har man då en bra känsel så kan man ju säga det men det är ju jättebra att du har ju fortfarande en bra känsel så att du behöver liksom inte medicinsk fotvård, man kan visa det och likadant tvärtom då, om dom verkligen har en dålig känsel att du nu behöver du en remiss till fotvården, så det är bra..	*If you have good sensation, then you can say it, but it's great that you still have good sensation so that you don't need medical foot care, you can show it and vice versa, if they really have reduced sensation and therefore need a referral to foot care, so that's good*
Jag dokumenterar när jag har patienten hos mig. Jag har också ett papper bredvid där jag också skriver lite anteckningar för att man vill ju gärna ha ögonkontakt också med patienten så att man har en dialog, så gör jag.	*I document when I have the patient with me. I also have a piece of paper next to it where I also write some notes, because you want to have eye contact with the patient so that you have a dialogue. I do that…*
…så jag känner väl det att det är ju jätteviktigt att formuläret är så enkelt att fylla i som möjligt, ja och att det ändå är liksom heltäckande på foten men ändå går snabbt att fylla i för man lägger ju mycket tid på dokumentation	*So I feel that it is very important that the form is as easy to fill in as possible and that it is still as comprehensive regarding the foot, but it can be filled in quickly because you spend a lot of time on documentation*
… många patienter ser ju faktiskt fram emot det här mötet och det är ju viktigt, att man, att det blir bra, ett bra möte och kanske att man kan ha en lättvindig dokumentation	*Many patients are actually looking forward to this meeting and it's important that it's a good meeting and that the documentation can perhaps be straightforward*

Jag har ju funderat lite på det och funderar ju på om det ska bli någon sån här slags kombibox, alltså att man kommer kunna välja alternativ som då faller ut i riskkategori ett och två och tre och fyra och sen om det blir åtgärder då sen som poppar upp per automatik?	*I have thought about it a bit and I wonder whether there shouldn't be some kind of combi box, i.e. that you are able to choose alternatives that then fall into risk category one, two, three and four and if there are then measures that pop up automatically*
Som vi har det nu så har vi ju papper när vi gör det och sedan för vi ju in det i journalen. Vi jobbar ju dubbelt nu, och är det så att det går att sköta allt i ett så är det ju lättare	*As it is now, we have paper when we do it and then we put it in the medical record. We do things twice now and, if it is possible to deal with everything once, it would be easier*
